# Unique binding modes for the broad neutralizing activity of single-chain variable fragments (scFv) targeting CD4-induced epitopes

**DOI:** 10.1186/s12977-017-0369-y

**Published:** 2017-09-22

**Authors:** Kazuki Tanaka, Takeo Kuwata, Muntasir Alam, Gilad Kaplan, Shokichi Takahama, Kristel Paola Ramirez Valdez, Anna Roitburd-Berman, Jonathan M. Gershoni, Shuzo Matsushita

**Affiliations:** 1 Matsushita Project Laboratory, Center for AIDS Research, Kumamoto University, 2-2-1 Honjo, Chuo-ku, Kumamoto, 860-0811 Japan; 2 Department of Cell Research and Immunology, George S. Wise Faculty of Life Sciences, Tel Aviv University, Tel Aviv, Israel

**Keywords:** HIV-1 neutralizing antibody, scFv, CD4i epitopes, Bridging sheet

## Abstract

**Background:**

The CD4-induced (CD4i) epitopes in gp120 includes the co-receptor binding site, which are formed and exposed after interaction with CD4. Monoclonal antibodies (mAbs) to the CD4i epitopes exhibit limited neutralizing activity because of restricted access to their epitopes. However, small fragment counterparts such as single-chain variable fragments (scFvs) have been reported to neutralize a broad range of viruses compared with the full-size IgG molecule. To identify the CD4i epitope site responsible for this broad neutralization we constructed three scFvs of anti-CD4i mAbs from a human immunodeficiency virus type 1 (HIV-1)-infected elite controller, and investigated the neutralization coverage and precise binding site in the CD4i epitopes.

**Results:**

We constructed scFvs from the anti-CD4i mAbs, 916B2, 4E9C, and 25C4b and tested their neutralization activity against a panel of 66 viruses of multi-subtype. Coverage of neutralization by the scFvs against this panel of pseudoviruses was 89% (59/66) for 4E9C, 95% (63/66) for 25C4b and 100% (66/66) for 916B2. Analysis using a series of envelope glycoprotein mutants revealed that individual anti-CD4i mAbs showed various dependencies on the hairpin 1 (H1) and V3 base. The binding profiles of 25C4b were similar to those of 17b, and 25C4b bound the region spanning multiple domains of H1 and hairpin 2 (H2) of the bridging sheet and V3 base. For 4E9C, the V3-base dependent binding was apparent from no binding to mutants containing the ΔV3 truncation. In contrast, binding of 916B2 was dependent on the H1 region, which is composed of β2 and β3 strands, because mutants containing the H1 truncation did not show any reactivity to 916B2. Although the H1 region structure is affected by CD4 engagement, the results indicate the unique nature of the 916B2 epitope, which may be structurally conserved before and after conformational changes of gp120.

**Conclusions:**

Identification of a unique structure of the H1 region that can be targeted by 916B2 may have an important implication in the development of small molecules to inhibit infection by a broad range of HIV-1 for the purpose of HIV treatment and prevention.

**Electronic supplementary material:**

The online version of this article (doi:10.1186/s12977-017-0369-y) contains supplementary material, which is available to authorized users.

## Background

Human immunodeficiency virus type 1 (HIV-1) entry into host cells is initiated by the interaction of CD4 of the host cell and gp120 of the viral envelope glycoprotein (Env). This interaction triggers conformational changes of gp120, exposing the co-receptor binding site on the surface of trimeric Env by the rearrangement of V1, V2 and V3 loops [1–5]. Then, the chemokine receptors bind to the bridging sheet region on gp120 and the entry step follows the membrane fusion through gp41 [6–9]. The bridging sheet consists of a four-stranded β-sheet structure composed of two double-strand β-sheet structures, hairpin 1 (H1 containing β2 and β3 in the stem of the V1/V2 loops) and hairpin 2 (H2 containing β20 and β21 in the C4 region) [5, 10]. These β-sheets are highly conserved among HIV-1 strains because the structure and amino acid sequences are critical for the interaction with the N-terminus of CCR5, suggesting it is the main component of the co-receptor binding site [11, 12]. The CD4-induced (CD4i) epitopes include the co-receptor binding site, which is formed and exposed after interacting with CD4.

Anti-CD4i neutralizing antibodies (nAbs) are frequently found in HIV-1-infected individuals and therefore the CD4i epitopes are regarded as an important target for antibody-mediated neutralization [13, 14]. However, most primary HIV-1 isolates are resistant to neutralization by anti-CD4i antibodies because the CD4i epitopes are hidden inside the trimeric Env before binding to CD4 [15]. Although the CD4i epitopes are exposed on the surface of trimeric Env after conformational changes induced by binding to CD4, it is still difficult for antibodies to access the target epitopes. The average size of IgG molecule is 115Å, but the space between the Env and target cell membrane is 45–80 Å after the engagement of CD4 to gp120. Therefore, the IgG is too big to access to the epitope due to the close physical proximity of gp120 to the cellular membrane [15]. The importance of the size required to access the exposed CD4i epitopes is demonstrated by improved neutralization using single-chain variable fragments (scFv). These small antibody fragments (~ 40 Å) constructed from anti-CD4i monoclonal antibodies (mAbs) neutralized primary viruses that were resistant to the parental IgGs. Furthermore, anti-CD4i scFvs neutralized viruses after CD4 binding [15–17].

The use of scFvs against the CD4i epitopes overcomes steric restriction and improves the neutralization potency against primary HIV-1 strains. The m9 scFv, which was selected from a random mutagenesis library of the scFv X5, neutralized approximately 80–90% of HIV-1 isolates of several subtypes [16, 17]. However, the 17b scFv, constructed from anti-CD4i Ab 17b [11], showed a poor neutralization potency [17]. The subtle difference in their reacting epitopes may account for the difference in neutralization potency among anti-CD4i scFvs. Analysis using a series of Env mutants revealed that individual anti-CD4i mAbs had various dependencies for the H1, V1/V2 loops and V3 stem regions [10, 18]. In addition, the binding of CD4 significantly affected the dependency on these regions [10, 18]. This suggests that anti-CD4i mAbs bind to the CD4i epitopes in various ways, although the structure of the CD4i epitopes is highly conserved.

We previously reported a series of mAbs targeting the CD4i epitopes from an HIV-1-infected elite controller [19]. In this study, we constructed small antibody fragments (scFvs and Fab) from three anti-CD4i mAbs, and analyzed their binding and neutralizing activities against HIV-1 isolates. These scFvs showed an effective and broad neutralization against a panel of HIV-1 strains from several subtypes. Especially, 916B2 scFv, which mainly recognized the H1 region, neutralized 100% (66/66 strains) of HIV-1 strains tested. These results indicated that the highly conserved CD4i epitopes, especially the H1 region, might be a vulnerable target for therapeutic agents.

## Methods

### Cells, reagents and viruses

TZM-bl [20–23], 293T [24], and 293A [25, 26], cells were maintained in Dulbecco’s modified Eagle’s medium (DMEM; Wako Pure Chemical Industries, Osaka, Japan) supplemented with 10% heat-inactivated fetal calf serum. Recombinant human soluble CD4 (sCD4) was purchased commercially (R&D Systems Inc., Minneapolis, MN, USA). Pseudoviruses were prepared by transfecting 293T cells with a plasmid expressing Env and env-deficient HIV-1 backbone vector pSG3ΔEnv, as previously described [19]. Env clones from a standard panel for subtypes A, B, C, D, A/D and A2/D were obtained from the NIH AIDS Reagent program [27–29]. Pseudovirus-containing culture supernatants were harvested 48 h post-transfection, filtered with 0.45-μm filters, and stored at − 80 °C until use.

### Construction of anti-CD4i antibodies

Monoclonal antibodies targeting the CD4i epitopes (916B2, 917B11, 4E9C, 5D6S, 25C4b, 12G10) were isolated from patient KTS376 infected with subtype B HIV-1 strains by B cell transformation, and recombinant IgGs were constructed, as previously described [19]. Briefly, RNA was extracted from a B cell line producing anti-CD4i IgG, and cDNA was synthesized using oligo(dT)20 and ReverTra Ace (Toyobo, Osaka, Japan). Immunoglobulin V regions were amplified by Platinum Taq DNA Polymerase High Fidelity (Invitrogen, Carlsbad, CA, USA). The IgG heavy chain and light chain κ expression plasmids were constructed by insertion of the PCR products into expression vectors, pIgGH and pKVA2, respectively. Fab heavy-chain expression plasmids were constructed by inserting the PCR product of heavy-chain into pHFab, which contains the heavy-chain CH1 region with HA-tag and His-tag. Light chain λ expression plasmids were constructed by inserting overlap extension PCR products into pcDNA3.1/Hygro (+). Light chain λ variable region, signal peptide region, and constant region were amplified using the cDNA sample, pLL-B404 [30], and pComb3Xlambda [31] as templates, respectively. Sequences of mAbs were aligned and phylogenetically analyzed using CLC Sequence Viewer 7 (CLC Bio, Aarhus, Denmark). The homologies between anti-CD4i mAbs were determined using Pairwise Sequence Alignment (EMBL Outstation-European Bioinformatics Institute, Wellcome Trust Genome Campus, Hinxton, Cambridgeshire, UK) [32].

Plasmids to express scFvs were constructed from IgG expression plasmids. The variable regions were amplified using the following primers: all three scFv VH: HSCVH1-FL (5′-GGT GGT TCC TCT AGA TCT TCC TCC TCT GGT GGC GGT GGC TCG GGC GGT GGT GGG CAG GTG CAG CTG GTG CAG TCT GG-3′) and scFvCH-R (5′-GTG ATG GTG ATG GTG GCC CTT GGT GGA GGC-3′), 916B2 scFv VL: scFv167BL-F (5′-AGA AGG AGA TAT ACA T ATG GCC TCC TAT GTG STG-3′) and HSCJLam1236-B (5′-GGA AGA TCT AGA GGA ACC ACC GCC TAG GAC GGT CAS CTT GGT SCC-3′), 4E9C scFv VK: scFvKDIV-F (5′-AGA AGG AGA TAT ACA T ATG GCC GAT ATT GTG WTG ACM CAG TCT CC-3′) and HSCJK2o-B (5′-GGA AGA TCT AGA GGA ACC ACC TTT GAT CTC CAG CTT GGT CCC-3′), and 25C4b scFv VK: scFvKDTT-F (5′-AGA AGG AGA TAT ACA T ATG GCC GAT ACG ACA CTC ACG-3′) and HSCJK2o-B. These PCR products were combined by overlapping PCR using scFv167BL-F and scFvCH-R for 916B2, scFvKDIV-F and scFvCH-R for 4E9C, and scFvKDTT-F and scFvCH-R for 25C4b. The PCR fragments were inserted into a pETHiH vector, a modified pETCF [33] with a *Stu*I site, a His-tag and an HA-tag at the 3′ region of the *Eco*RI site, after digestion with *Nde*I and *Stu*I using GeneArt Seamless Cloning and Assembly kit (Invitrogen).

### Expression and purification of anti-CD4i antibodies

Recombinant IgG and Fab were produced and purified as previously described [19]. Briefly, heavy and light chain plasmids were transfected to 293A cells and cells stably expressing recombinant IgG or Fab were selected with G418 (1000 μg/ml) and Hygromycin (150 μg/ml). IgG proteins were purified using a HiTrap rProtein A FF Column (GE Healthcare, Buckinghamshire, UK). Fab proteins were purified using a His 60 Ni Superflow Resin column (Clontech, Mountain View, CA, USA) via a His-tag attached to a heavy chain expression plasmid.

Anti-CD4i scFv proteins were produced in *Escherichia coli* Rosetta 2 (DE3) (Merck, Darmstadt, Germany) transformed by a scFv expression plasmid, as described previously [33]. Briefly, the transformed cells were cultured in Luria–Bertani (LB) broth containing 100 µg/ml carbenicillin, and production of scFv was induced with 2 mM isopropyl-b-d(−)-thiogalactopyranoside (IPTG, Wako Pure Chemical Industries). The inclusion body was solubilized in PBS containing 6 M guanidine hydrochloride (Gu-HCl, Wako Pure Chemical Industries), and scFv protein was purified using a His 60 Ni Superflow Resin column (Clontech). The eluted scFv protein was refolded in Spectra/Por dialysis membrane (Spectrum Laboratories, Rancho Dominguez, CA, USA) by dialysis with a gradient Gu-HCl concentration. Purified antibody proteins were analyzed by SDS-PAGE using Extra PAGE One Precast Gel 5–15% (Nacalai Tesque, Inc., Kyoto, Japan) and Bio-Safe Coomassie blue G-250 (Bio-Rad, Hercules, CA, USA).

### Binding activity of antibody fragments against monomeric gp120

Binding activity of anti-CD4i mAbs was determined by gp120 capture ELISA. A polyvinyl chloride flexible 96 well plate (BD Falcon, Franklin Lakes, NJ, USA) was coated with 50 µl of 10 μg/ml anti-gp120 sheep polyclonal antibody D7324 (Aalto BioReagents, Dublin, Ireland) overnight at 4 °C. HIV-1 gp120 was conjugated by the addition of 50 μl of 1 μg/ml SF2 gp120 (kindly supplied by Emi Nakayama, Department of Viral Infections, Osaka University, Japan), and subsequent incubation overnight at 4 °C. After incubation, 100 µl of Abs was added to each well in the presence or absence of 1 μg/ml soluble CD4 (sCD4). IgG binding was detected by 100 µl of Anti-Human IgG (γ-chain specific)-Alkaline Phosphatase goat antibody (1:2000 dilution, Sigma, St. Louis, MO, USA). Fab and scFv binding were detected by 100 µl of anti-HA High Affinity (0.1 µg/ml, 3F10, Roche Molecular Biochemicals, Mannheim, Germany) and 100 µl of Goat Anti-Rat IgG H&L (Alkaline Phosphatase) (1:2500 dilution, Abcam, Cambridge, UK). Finally, phosphatase substrate (Sigma) was added, and the absorbance at 405 nm was measured using a microplate reader (Bio-Rad).

### Binding activity of antibody fragments to HIV-1 Env by flow cytometry analysis

Binding activity of IgG, Fab and scFv was determined using 293T cells transfected with a plasmid expressing both Env from the JR-FL strain and enhanced green fluorescent protein (EGFP). Transfected cells were detached with PBS containing 0.05% trypsin and 0.53 mM EDTA and adjusted to 1 × 10^7^ cells/ml in PBS with or without 2 μg/ml sCD4. After incubation for 15 min at RT, 20-μl cells were incubated with 10 μl of 6 μg/ml antibody for 20 min at RT. IgG binding was detected by allophycocyanin-conjugated AffiniPure F(ab′)2 Fragment Goat Anti-Human IgG (H+L) (1:200 dilution, Jackson ImmunoResearch, West Grove, PA, USA), and the binding of Fab and scFv was detected by anti-HA High Affinity (1:200 dilution) and allophycocyanin-conjugated AffiniPure F(ab′)2 Fragment Goat Anti-Rat IgG (H + L) (1:200 dilution, Jackson ImmunoResearch). Finally, Ab binding was detected by FACS CantoII (Becton–Dickinson, Franklin Lakes, NJ, USA). Data analysis was performed using FlowJo software (TreeStar, San Carlos, CA, USA).

### Determination of neutralization activity

#### Standard neutralization assay

The neutralization activities of the IgGs and antibody fragments of anti-CD4i mAbs were evaluated using TZM-bl cells, as described previously [19]. Briefly, serial dilutions of antibody and virus (400 TCID_50_) were pre-incubated at 37 °C for 1 h in 96-well tissue culture plates. The TZM-bl cell suspension containing DEAE-Dextran (25 µg/ml) in DMEM with 10% FCS was added to each well. After incubation for 48 h at 37 °C with 5% CO_2_, the wells were washed once with PBS and lysed with lysis buffer (Life Technologies, Carlsbad, CA, USA). The lysate was transferred to an opaque white plate containing-galactosidase substrate (Life Technologies) and incubated for 1 h with protection from light. The galactosidase activity was measured using a Centro XS3 LB960 luminometer (Berthold Technologies, Bad Wildbad, Germany). The reduction in infectivity was determined by comparing the relative light units (RLU) in the presence and absence of antibody and was expressed as a percentage of neutralization. The half-maximal (50%) inhibitory concentration (IC_50_) was calculated using nonlinear regression and defined as the concentration that caused a 50% reduction in luciferase activity.

#### Pre-attachment neutralization assay

The pre-attachment neutralization activities of the IgGs and scFvs were evaluated using TZM-bl cells and pseudo-virus expressing mouse CD4 on the viral surface as previously reported [34]. First, 1 ml of pseudo-virus corresponding to the titer yielding about 200,000 RLU was incubated with 10 µl mouse CD4 (L3T4) MicroBeads (Miltenyi Biotec, Bergisch Gladbach, Germany) for 30 min at 4 °C. The virus-bead complexes were bound to μMACS columns (Miltenyi Biotec), and washed and eluted with 1 ml of DMEM containing 10% FCS. The virus-bead complexes were incubated with equal volume of 200 μg/ml scFvs for 1 h at RT. Parental IgGs, b12 mAb targeting CD4bs and KD-247 mAb targeting V3-loop were used for the comparison with scFvs [35, 36]. After incubation, the complexes were bound to the columns again, and then unbound Abs were removed by washing. The complexes were eluted with DMEM containing 10% FCS. TZM-bl cells and the virus-Ab complexes were added with 25 µg/ml DEAE-Dextran to a 96-well culture plate. Thereafter, the incubation and detection were performed similarly to the standard neutralization assay. The detected RLU values were normalized by the amount of input virus, which was determined with a p24 ELISA kit (ZeptoMetrix Corporation, Buffalo, NY, USA).

#### Temperature-regulated neutralization (TRN) assay

The post-attachment neutralization activities of anti-CD4i Abs were measured using a temperature-regulated neutralization (TRN) assay as described previously [15, 33]. Briefly, 100 μl of TZM-bl cells were plated at 2 × 10^4^ cells per well in 96-well cell culture plates and incubated at 37 °C overnight for attachment. Culture medium was replaced with 150 μl of cold growth medium and 50 μL of pseudoviruses corresponding to a titer yielding about 200,000 RLU with DEAE-Dextran (25 µg/ml). After incubation at 4 °C for 1 h, unbound viruses were removed by washing twice with cold medium, and 150 μl of cold growth medium was added. Finally, 50 μL of serially diluted Abs in cold growth medium were added to the wells, and the temperature was increased to 37 °C. Thereafter, the incubation and detection were performed similarly to the standard neutralization assay.

### Binding properties of anti-CD4i mAbs to BaL mutants

The binding profiles of anti-CD4i mAbs against monomeric gp120 were analyzed by capture ELISA using the gp120s of BaL wild-type (WT) and mutants as previously reported [10, 37]. WT and mutant BaL gp120s, V3 base, ΔV3, ΔV1V2, ΔV1, ΔV2, ΔH1, ΔH1–V3 base and ΔH1–ΔV3 were produced in transfected 293T cells, and the culture medium was collected after 48 h post-transfection and filtered with 0.45-μm filters. The amount of gp120 was quantified by gp120 capture ELISA using anti-CD4bs mAb 49G2, and SF2 gp120 as a standard. An ELISA plate was coated with 50 μl of 10 μg/ml D7324 overnight at 4 °C. After blocking with 2% BSA in PBS at 37 °C for 1 h, 50 µl of 5 μg/ml mutant gp120 was added to the wells. After incubation at 37 °C for 1 h, 100 µl of 2.5 μg/ml mAbs was added to each well in the presence or absence of sCD4 (4 μg/ml). After incubation at 37 °C for 1 h, peroxidase-conjugated AffiniPure F(ab′)2 Fragment Goat Anti-Human IgG (H+L) (1:2500 dilution; Jackson ImmunoResearch) was added to each well, and incubated at RT for 1 h. Finally, the wells were reacted with 100 µl ABTS [2,2′-azinobis-(3-ethylbenzthiazoline sulfonic acid)] solution (Roche Molecular Biochemicals) and the absorbance at 405 nm was measured using a microplate reader. All binding assays were performed at least twice and representative results are shown.

## Results

### Construction of antibody fragments against the CD4i epitopes of gp120

**Fig. 1:**
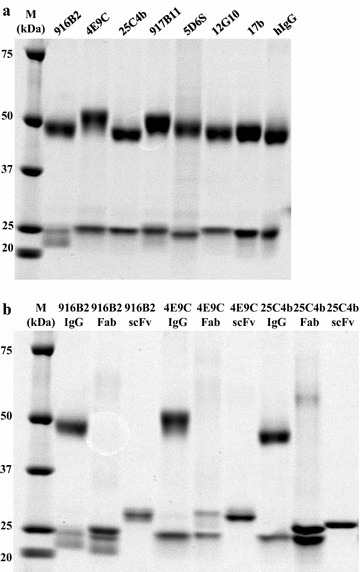
SDS-PAGE analysis of purified IgGs and antibody fragments targeting CD4i epitopes. Purified IgG of each mAb against the CD4i epitopes (**a**), and IgG, Fab and scFv of three mAbs, 916B2, 4E9C and 25C4b (**b**), were analyzed by SDS-PAGE. IgG was purified from the culture supernatant of transformed B cells or transfected 293A cells with a Protein A column. Fabs were purified from the culture supernatants of transfected 293A cells with a Ni column. ScFvs from *E. coli* strain Rosetta 2 (DE3) transformed cells were purified with a Ni column and refolded. M, size marker

In this study, six anti-CD4i mAbs, 916B2, 4E9C, 25C4b, 917B11, 5D6S and 12G10 (including 4 reported previously) were cloned from an HIV-1 infected patient, KTS376 [19]. Immunoglobulin variable regions amplified from Epstein-Barr Virus-immortalized cell lines producing anti-CD4i mAbs were cloned into IgG expression vectors, and 916B2, 4E9C and 25C4b were used for the construction of Fab and scFv. The IgG and Fab proteins were produced in 293A cells, and scFv proteins were expressed in *E. coli*. The respective IgG and antibody fragments were purified using protein A- or Ni-column chromatography (Fig. 1). The sizes of heavy chain fragments were different among anti-CD4i mAbs. The heavy chains of 4E9C IgG and Fab were larger than that of the other antibodies. However, the size of light chains was similar between all mAbs except 916B2, which had a smaller counterpart in addition to a light chain of normal size. 916B2, 4E9C and 25C4b scFvs had a molecular weight of approximately 30 kDa (Fig. 1b).

To analyze the genetic characteristics of these clones, their immunoglobulin genes were sequenced and compared. As listed in Additional file 1: Table S1, all cloned antibodies except 25C4b utilized the VH1-69 gene, which most anti-CD4i antibodies preferentially use [38–40]. A long CDRH3 with multiple tyrosine residues, another feature of Abs against the CD4i epitopes [38, 41, 42], was observed in 5 of 6 mAbs. In contrast, 916B2 had a shorter CDRH3 (16 aa) compared with the other mAbs and only a single tyrosine in CDRH3. These results suggest the preferential usage of the VH1-69 gene and long CDRH3 with tyrosine residues in the heavy chain are significant features of anti-CD4i mAbs from patient KTS376, similar to previous reports [38–42].

In addition, 4E9C and 12G10 belong to the same lineage of Abs because of their identical gene usage and high sequence homology. However, the size of the heavy chain of 4E9C was larger than that of 12G10 (Fig. 1) and might be because of differences in post-translational modifications, such as phosphorylation, glycosylation or sulfation.

### Broad neutralization of the panel of pseudoviruses by anti-CD4i scFvs

**Fig. 2:**
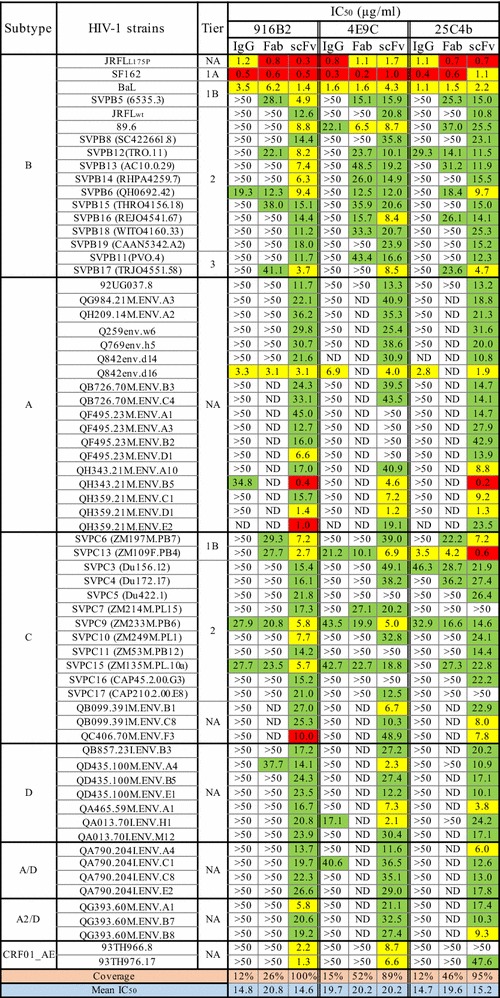
Neutralization activities of IgGs and antibody fragments against multi subtype HIV-1 strains. The neutralization activities of IgGs and antibody fragments of anti-CD4i mAbs were measured using TZM-bl cells and single-round infection assay, and shown as the IC_50_ (μg/ml). The IC_50_ values were highlighted by the following colors: red; 0.1–1.0 μg/ml, yellow; > 1.0–10 μg/ml, green; > 10–50 μg/ml. NA: Not applicable. ND: Not done

A standard single-round neutralization assay was performed to compare the neutralization activities of the recombinant antibody fragments from three mAbs, 916B2, 4E9C and 25C4b, with the corresponding IgGs (Fig. 2).

As expected from previous studies [19], the IgG form of these mAbs neutralized a few HIV-1 strains. Of the subtype B strains, only 3 neutralization-sensitive strains, JR-FL_L175P_, SF162 and BaL, were neutralized by all the IgGs. JR-FL_L175P_ has a mutation at amino acid residue 175 that changes JR-FL WT to the neutralization sensitive phenotype [43], and SF162 and BaL were categorized to very high (tier 1A) and above-average (tier 1B) neutralization sensitivities [44]. Most neutralization-resistant tier 2 and 3 viruses of subtype B were resistant to these IgGs. In contrast to IgGs, the corresponding scFvs neutralized all 17 subtype B strains tested. The Fabs of 916B2, 4E9C and 25C4b neutralized 8, 13 and 10 subtype B strains, respectively, of 17 strains in total, indicating a moderate neutralization potency between those of IgG and scFv.

The neutralization coverage of the scFvs was also remarkable against panels of non-subtype B viruses (subtype A, A/D, A2/D, C, D and CRF01_AE). The neutralization coverage of 4E9C and 25C4b scFvs was 89% (59/66) and 95% (63/66) of all viruses, respectively. Moreover, 916B2 scFv showed 100% (66/66) coverage of multi-subtype strains tested (Fig. 2). The Fabs were less effective against non-subtype B viruses with from 26 to 52%, which was greater than that of IgG (12–15%).

These results are consistent with previous observations that suggested an improvement in the neutralization of anti-CD4i nAbs by reducing the molecule size [15–17]. Moreover, 916B2 scFv neutralized a broad range of HIV-1 strains.

#### Mechanism of neutralization of scFvs, Fabs and their IgG counterparts against the CD4i epitopes

**Fig. 3:**
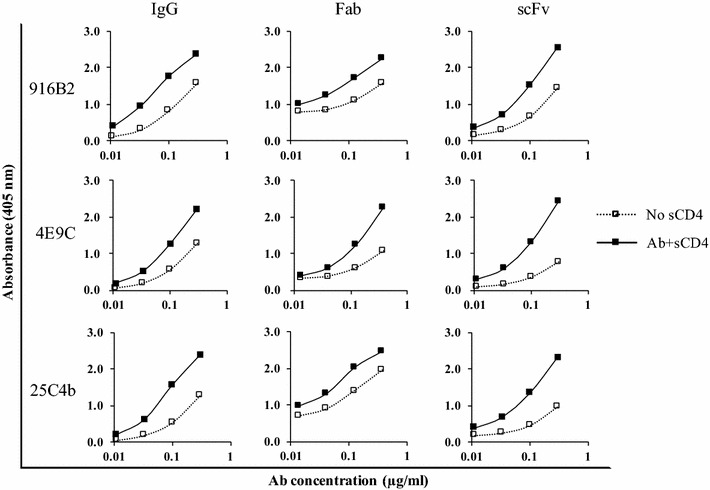
Binding of antibody fragments from anti-CD4i mAbs to the monomeric gp120. Binding activities of IgG and antibody fragments of each mAb to monomeric gp120 of SF2 were examined in the presence (solid square) or absence (open square) of sCD4 by capture ELISA. Binding activity of anti-CD4i mAbs was enhanced by sCD4

**Fig. 4:**
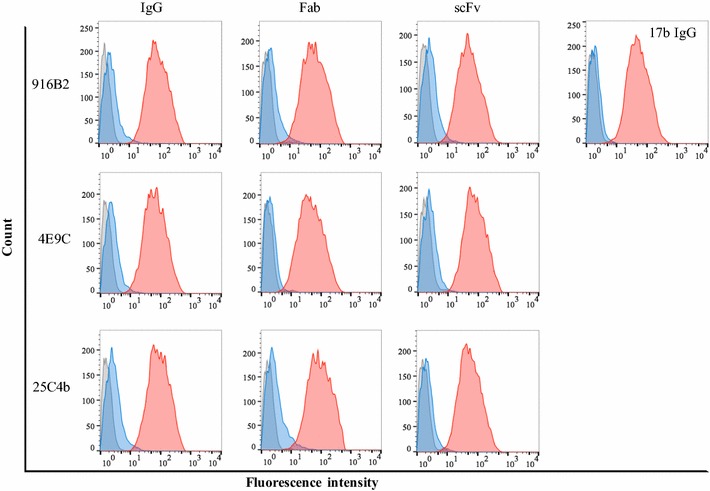
Binding enhancement of mAbs and their fragments to Env in the presence of sCD4. Binding activity to 293T cells expressing Env of JR-FL was examined using flow cytometry. Histogram of fluorescence intensity shows the binding of negative control (gray), mAb alone (blue) and in the presence of sCD4 (red). Enhancement of binding by CD4 engagement against trimeric Env expressed on the cellular surface was observed for anti-CD4i IgG as well as their fragments

The binding activities of the anti-CD4i antibody fragments against monomeric gp120 and trimeric Env were determined using gp120-capture ELISA and flow cytometric analyses, respectively [19]. The enhancement of binding to monomeric gp120 in the presence of sCD4 was observed for IgG as well as Fab and scFv (Fig. 3 and Additional file 2: Table S2). Binding against trimeric Env expressed on the cellular surface was examined by flow cytometry using 293T cells expressing Env (Fig. 4 and Additional file 3: Fig. S1). Enhancement of binding by CD4 engagement against trimeric Env was observed for anti-CD4i IgGs as well as their Fab and scFv forms. These results suggest that CD4 binding to gp120 is important to induce the correct conformation of the epitopes of the anti-CD4i mAbs including small antibody fragments.

**Fig. 5:**
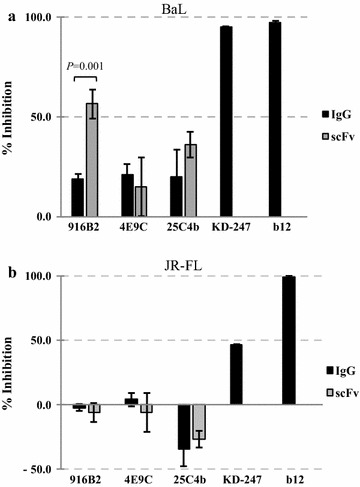
Pre-binding neutralization activity. The neutralization activities of anti-CD4i mAbs against BaL (**a**) and JR-FL (**b**) before the attachment of virus and target cell were analyzed. The pseudo-virus infected the target cells after pre-incubation in the presence of the neutralizing mAbs. Anti-CD4bs mAb, b12 (10 μg/ml), anti-V3 mAb, KD-247 and anti-CD4i mAbs (100 μg/ml) were used. The neutralization activities of the IgGs (black) and scFvs (gray) were standardized by the amount of p24

To identify which step in infection is critical for neutralization by scFv, pre-attachment neutralization assays and post-attachment neutralization assays were performed. Although the pre-attachment neutralization activity was low for all anti-CD4i IgGs and scFvs compared with b12 targeting the CD4bs epitope, the 916B2 scFv showed greater than 50% pre-attachment neutralization activity against the BaL strain, which was significantly greater than that of the corresponding IgG (Fig. 5a, *P* = 0.001). In contrast, significant improvement of pre-attachment neutralization potency was not observed in the 4E9C and 25C4b scFv against BaL, and all the scFvs against JR-FL (Fig. 5). Compared with BaL (tier 1B), JR-FL (tier 2) was resistant to pre-attachment neutralization, as shown by nearly 100% inhibition of BaL and about 50% inhibition of JR-FL by anti-V3 mAb KD-247. The trimeric Env structure of BaL, which allows the access of anti-V3 Ab, may be important for the improvement of pre-attachment neutralization activity of anti-CD4i scFv.

**Fig. 6:**
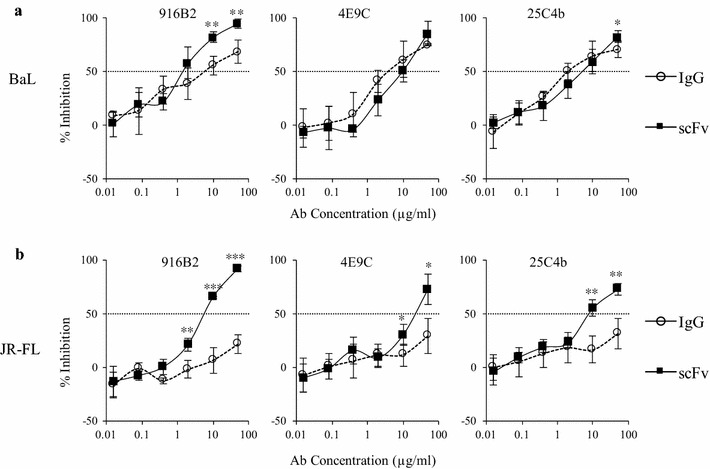
Post-binding neutralization activity. The neutralization activities of anti-CD4i mAbs against BaL (**a**) and JR-FL (**b**) after attachment of virus and target cell were analyzed. The neutralizing activities of IgGs are shown as open circle, and scFvs are shown as closed square. scFvs targeting the CD4i epitopes inhibited virus infection after virus-attachment to the target cells against both viruses. The statistical significance was calculated using a Student’s *t* test (*P < 0.05, **P < 0.005, ***P < 0.0005)

Both the IgG and scFv of the 3 anti-CD4i mAbs showed post-attachment neutralization activity against BaL (Fig. 6a). The efficient neutralization of BaL by all the anti-CD4i IgGs after the attachment of viruses to cells, but no neutralization by the IgGs before attachment, suggests that post-attachment neutralization is the main inhibitory mechanism of anti-CD4i IgGs against neutralization-sensitive strains. The level of inhibition against BaL strain by each scFv was similar to the corresponding IgG, but the 916B2 scFv showed slightly greater inhibition than the IgG. However, post-attachment neutralization of the JR-FL strain, which was resistant to neutralization by anti-CD4i IgGs, was significantly different between the IgG and scFv (Fig. 6b). All three anti-CD4i IgGs did not neutralize the JR-FL strain, but all the scFvs induced greater than 50% inhibition. These results suggest that anti-CD4i scFvs effectively neutralize a broad range of HIV-1 strains by a post-attachment neutralizing mechanism.

#### Multiple binding profiles of anti-CD4i mAbs

**Fig. 7:**
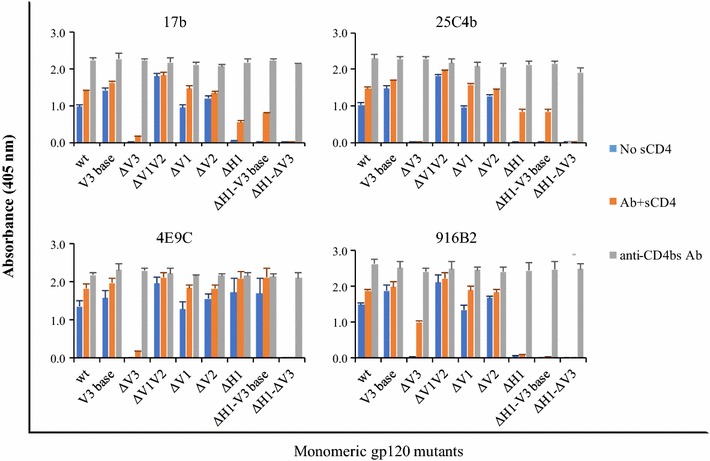
Binding activities of anti-CD4i mAbs against gp120 mutants. The binding activities of anti-CD4i mAbs against gp120 from BaL WT and mutants were measured by gp120 capture ELISA. We used the monomeric gp120 of WT and eight mutants including V3 base (gp120 with truncated V3 loop tip), ΔV3 (gp120 with truncated V3 loop containing the base of V3), ΔV1V2 (gp120 with truncated V1 and V2 loops), ΔV1 (gp120 with truncated V1 loop), ΔV2 (gp120 with truncated V2 loop), ΔH1 (gp120 with truncated V1/V2 loops containing the stem of the loops), ΔH1–V3 base (gp120 with the deletions of ΔH1 and V3 base mutants) and ΔH1–ΔV3 (gp120 with the deletions of ΔH1 and ΔV3) as previously described [10]. The binding activities of anti-CD4i mAbs in the presence (orange) or absence (blue) of sCD4 and anti-CD4bs mAb, 49G2 (gray) are shown

The binding profiles of anti-CD4i mAbs were analyzed by capture ELISA using the gp120 of BaL WT and a series of mutants to identify the region in gp120 important for broad neutralization (Fig. 7). We observed that 25C4b bound to the ΔH1 mutant (truncation of V1/V2 loops and the stem) and ΔH1–V3 base (truncation of hairpin 1 and a partial V3 loop) in the presence of sCD4, while no binding was observed for the ΔH1–ΔV3 double mutant (truncation of hairpin 1 and a full V3 loop). Although the marginal recovery of binding to the single mutant ΔV3 (truncation of V3 loop) with sCD4 was not observed for 25C4b, the binding profiles of 25C4b were similar to those of 17b. This suggests that 25C4b binds a region spanning multiple domains of H1 and H2 of the bridging sheet and V3 base.

4E9C showed V3-base dependent binding because it did not bind to mutants containing the V3-base truncation. Binding of 4E9C was observed in all the mutants except for those containing the ΔV3 mutation even in the absence of sCD4. The marginal recovery of 4E9C binding observed for the ΔV3 mutant in the presence of sCD4 indicated that part of the 4E9C epitope was formed using a region such as H2 after interacting with gp120 and sCD4.

The binding of 916B2 was dependent on the H1 region, because mutants containing the H1 truncation did not show any reactivity to 916B2. The recovery of binding of 916B2 against the ΔV3 mutant by the engagement of sCD4 was greater compared with 4E9C and 17b, suggesting that truncation of the V3-base was compensated by the structure after interacting with gp120 and sCD4. These results imply that the H1 region composed of β2 and β3 structures may be an important target for 916B2 scFv, which neutralizes a broad range of HIV-1 strains.

We also determined the binding profiles of 3 additional CD4i mAbs together with A32 targeting the C1 region of gp120 (Table 1 and Additional file 4: Fig. S2) [45, 46]. The binding profile of 12G10 was similar to 25C4b and 17b, in which both H1 and H2 are responsible for binding. Although the binding of 917B11 was enhanced by the engagement of sCD4 compared with 4E9C, the profile of 917B11 was similar to that of 4E9C, which showed V3 base-dependent binding. The binding profile of 5D6S was different from the other anti-CD4i antibodies. Its binding to gp120 was dependent on the HI and V3 regions in the absence of sCD4. However, we observed the recovery of binding to all the mutants in the presence of sCD4, suggesting the recognition of H1, H2 and V3 in the CD4 bound form of the bridging sheet. Taken together, these results suggest multiple modes of recognition by antibodies to the CD4i epitopes, which are composed of a bridging sheet containing H1, H2 and the base of V3 (Additional file 5: Fig. S3). The structure formed by the arrangement of these domains after the conformational change induced by sCD4 also contributes to the variety of antibody recognition. Interestingly, the mAbs 4E9C and 12G10, which belong to the same linage and are genetically homologous, had different binding profiles.

**Table 1:** Summary of anti-CD4i mAb binding to different gp120 mutants

Protein	Binding							
	916B2	17b	25C4b	12G10	4E9C	917B11	5D6S	A32
wt								
Absence of sCD4	+++	+++	+++	++	+++	+++	++	++
Presence of sCD4	+++	+++	+++	+++	+++	+++	+++	++
V3 base								
Absence of sCD4	+++	+++	+++	+++	+++	+++	+++	+++
Presence of sCD4	+++	+++	+++	+++	+++	+++	+++	+++
ΔV3								
Absence of sCD4	−	−	−	−	−	−	+	+
Presence of sCD4	+++	+	−	−	+	++	+++	++
ΔV12								
Absence of sCD4	+++	+++	+++	+++	+++	+++	+++	+++
Presence of sCD4	+++	+++	+++	+++	+++	+++	+++	+++
ΔV1								
Absence of sCD4	+++	+++	+++	++	+++	+++	++	++
Presence of sCD4	+++	+++	+++	+++	+++	+++	+++	+++
ΔV2								
Absence of sCD4	+++	+++	+++	+++	+++	+++	+++	+++
Presence of sCD4	+++	+++	+++	+++	+++	+++	+++	+++
ΔH1								
Absence of sCD4	−	−	−	−	+++	++	+	+++
Presence of sCD4	−	++	++	++	+++	+++	+++	+++
ΔH1–V3 base								
Absence of sCD4	−	−	−	−	+++	++	−	+++
Presence of sCD4	−	++	++	++	+++	+++	++	+++
ΔH1–ΔV3								
Absence of sCD4	−	−	−	−	−	−	−	++
Presence of sCD4	−	−	−	−	−	−	+++	++

Relative binding activities of anti-CD4i mAbs against each gp120 mutant with the binding of anti-CD4bs Ab

* −: < 0.05, +: > 0.05–0.2, ++: > 0.2–0.4, +++: > 0.4

## Discussion

We constructed three scFvs from a panel of mAbs targeting the CD4i epitopes and showed the broad neutralizing activity of anti-CD4i scFvs. These scFvs neutralized most viruses belonging to multiple subtypes, many of which were not neutralized by their IgG forms. Especially, 16B2 scFv showed 100% coverage of all viruses tested. This neutralization coverage is much higher than scFv m9, which was previously reported to be comparable to broadly neutralizing antibodies, though the coverage was not simply compared because of the difference of HIV-1 strains used [17]. Antibodies against the CD4i epitopes recognized a conserved region of gp120 that overlaps with the co-receptor binding site. However, neutralization potency was restricted by adjacent variable loops and steric and conformational constraints [15]. In line with a previous report by Labrijin et al. [15], broad neutralization by these scFvs is attributable to the post-attachment neutralization mechanism (Fig. 6). Previous studies that analyzed the binding of CD4i mAbs to gp120 by crystallographic structure [38, 46–53] and performed binding analysis using a series of gp120 mutants [10–12, 18], suggested multiple interactions of CD4i mAbs with H1 and H2 of the bridging sheet and the base of V3 for binding. In this study, we also observed multiple modes of recognition by CD4i-antibodies against epitopes formed by the component of bridging sheet and the base of V3.

Our data suggest the main target for 25C4b and 12G10 resembles 17b, which interacts with H1, H2 and requires the V3 base [10]. However, 4E9C and 917B11 had more interactions with H2 and V3 base and have similarity with 412d as determined by crystallographic analysis [51]. Interestingly, 916B2 appeared to have unique binding feature that was H1 dependent. The characteristic of H1-dependent binding was similar to 21c binding [10], although 21c requires a V1V2 loop in the absence of sCD4. This profile suggests that 21c equivalently binds to H1 and H2 and requires interaction with the V2 core sites in the absence of sCD4. Interactions of gp120 with CD4 changes the binding mode, and 21c binds to the bridging sheet at the physical juxtaposition of H1 and H2 via the alignment of β2 with β21 in the presence of sCD4 [10]. In addition, the binding of 21c to CD4 in the absence of gp120 was reported together with the importance of the V1V2 loop for the binding of the mAb [53]. Recognition of the H1, H2 and V3 base regions, but independent of the V1V2 loop, may account for the broad neutralizing potency of the 916B2 scFv.

The H1 region, which is the main target of 916B2, is located at the base of the V1V2 loop, and consists of β2 and β3 of the bridging sheet. In the absence of sCD4, H1 becomes folded and hydrogen bonds might form between the β3 of H1 and β21 of the H2 [10, 54]. After engagement with CD4, the tip of H2 bends to its bottom side and the hydrogen bonds of β3–β21 are interrupted, and then the H1 fully extends and twists to switch the positions of β2 and β3 followed by hydrogen bonding between β2 and β21 [10, 54]. From our data of 916B2 binding against gp120 mutants, the epitope recognized by 916B2 is the H1 region, which consists of β2 and β3 strands in juxtaposition. For the ΔV3 mutant with a truncated base of V3, 916B2 binding was not observed without sCD4. This phenomenon may be explained by a change in H1 structure, which was indirectly caused by the deletion of the base of V3. The deletion of V3 affects the conformation of H2, which results in dislocation of the β3 strand from the β2 of H1 because of the hydrogen bonds between the β3 and β21 [55–57]. In contrast, the shift and reorientation of the β21 strand induced by sCD4 binding disrupted the β3–β21 interaction and fully exposed the extended H1. We observed the recovery of 916B2 binding to the ΔV3 mutant in the presence of sCD4. Taken together, these results support the sequential conformational changes of the bridging sheets by CD4 binding and suggest portions of the epitope of 916B2 is structurally conserved before and after the conformational changes of gp120. Consistently, although 916B2 mAb bound Env of JR-FL strain, which only the scFv neutralized, on the cell surface strongly in the presence of sCD4, 916B2 mAb bound the surface anchored Env of BaL strain even in the absence of sCD4 (Fig. 4 and Additional file 3: Fig. S1). Moreover, the BaL strain was neutralized by all three forms of 916B2 (Fig. 2) and the scFv showed considerable pre-attachment neutralization activity (Fig. 5). These results indicate that the 916B2 epitope is also conserved before and after CD4 binding on the surface of cell and virion.

The mAbs 25C4b and 12G10 showed similar binding profiles with 17b. However, in contrast to 17b binding, binding activities to the ΔV3 mutant of these mAbs were not recovered by adding sCD4. Thus, the deletion of H1 changes 25C4b and 12G10 binding which is absolutely CD4-dependent. The potential for these mAbs to interact with the base of V3 remain to be elucidated. The binding profiles of 4E9C and 917B11 to the mutant with fully truncated V3 with its base were similar to that of 17b. Surprisingly, 4E9C and 917B11 bound the ΔH1 mutant without CD4 binding. Moreover, the binding activities of these mAbs to the ΔH1 mutant were comparable to the ΔV1V2 mutant. Thus, 4E9C and 917B11 interact with H2 and the binding depends on the base of V3. The phylogenetic analysis of 4E9C and 12G10 suggested these two antibody-producing cells have a common ancestor (Additional file 6: Fig. S4). However, the binding profile of 12G10 is different from 4E9C and resembles that of 17b and 25C4b. The shift of antibody recognition within the bridging sheet suggests a different maturation pathway of B-cells in the same B cell lineage. Interestingly, we observed binding activity to ΔH1–ΔV3 mutants for 5D6S in the presence of sCD4. No other anti-CD4i mAbs has been reported to bind to this mutant to date [10]. It is conceivable that the main target of 5D6S is H2 especially in its CD4-bound form, and this is unique for CD4i mAbs.

Most anti-CD4i mAbs utilize the VH1–69 for the V-gene of the heavy chain, have a long CDRH3, and frequently use acidic amino acids together with sulfated tyrosines, referred to as molecular mimicry of the N-terminal region of CCR5 [38, 42, 51, 58]. The genetic features of anti-CD4i mAbs in the present study, except for 916B2, are consistent with previous studies. Moreover, these mAbs were found to have the “DYYD” or “DYYE” motifs in CDRH3 and shared binding to the H2 region (Fig. 7, Additional file 4: Fig. S2 and Additional file 1: Table S1). A study of broadly neutralizing mAbs targeting the V1V2 loops reported the utilization of these motifs and the formation of a long protruding loop [59, 60]. Therefore, the CDRH3s of the mAbs in the current study with these motifs in the CDRH3 might form a protruding loop and interact with the H2 region.

Various modes of binding were observed for a series of anti-CD4i mAbs over the bridging sheet and related regions of gp120. Identification of a structure in the H1 region that is targeted by 916B2 may have important implications for the development of small molecules to inhibit the infection of a broad range of HIV-1 for the purpose of treatment and prevention.

## Conclusions

We constructed the scFvs of anti-CD4i mAbs from an HIV-1-infected elite controller, and observed the broad neutralization of the scFvs against HIV-1 strains belonging to multi-subtypes. Of note, 916B2 scFvs neutralized all pseudoviruses tested. The binding of 916B2 is dependent on the H1 region, which is composed of β2 and β3 strands. Although the H1 region structure is susceptible to conformational change by CD4 engagement, the results indicate that 916B2 targets a structure in the H1 region, which is conserved before and after CD4 binding. This highly conserved H1 region might be a vulnerable target for therapeutic agents.

## Additional files

**Additional file 1: Table S1.** Sequence profile of cloned anti-CD4i mAbs.

**Additional file 2: Table S2.** Binding activity of anti-CD4i antibodies against monomeric gp120.

**Additional file 3: Figure S1.** Binding enhancement of mAbs and their fragments to Env of BaL strain in the presence of sCD4. Binding activity to 293T cells expressing Env of BaL was examined using flow cytometry. Histogram of fluorescence intensity shows the binding of the mAbs as described in Fig. 4.

**Additional file 4: Figure S2.** Binding activities of other anti-CD4i mAbs against gp120 mutants. The binding activities of other anti-CD4i mAbs against gp120 were measured by gp120 capture ELISA. We used the monomeric gp120 of WT and eight mutants as described in Fig. 7. The binding activities of anti-CD4i mAbs in the presence (orange) or absence (blue) of sCD4 and anti-CD4bs mAb, 49G2 (gray) are shown.

**Additional file 5: Figure S3.** Comparison of the epitopes of anti-CD4i mAbs. The regions of anti-CD4i mAbs interaction sites were highlighted with each color. The H1 (cyan), H2 (pink), the base of V3 (yellow) and the tip of V3 (green) are shown (PDB accession number 2B4C). 25C4b interacts a region spanning multiple domains of H1 and H2 of the bridging sheet and V3 base as 17b and 412d bindings. 4E9C and 916B2 show the signature bindings against gp120, respectively.

**Additional file 6: Figure S4.** Phylogenetic analysis of the variable region sequence of 4E9C and 12G10. Variable region sequences of heavy- and kappa-chains were aligned and phylogenetically analyzed. The sequences of anti-CD4i mAbs utilizing IGHV1–69 (412d, 23e, 47e, E51, X5), IGHV1–24 (411g, 48d, 16c), IGKV3–15 (411g) and IGKV3–20 (X5) were obtained from GenBank. The homologies of immunoglobulin genes of heavy- and kappa-chains between anti-CD4i mAbs were determined using Pairwise Sequence Alignment. The same gene usage and high sequence homology between 4E9C and 12G10 are shown (green): 86% for heavy chain and 93% for kappa chain.
