# Supplementary figures and images for: Unique binding modes for the broad neutralizing activity of single-chain variable fragments (scFv) targeting CD4-induced epitopes
Source: Retrovirology. 2017 Sep 22;14:44. doi: 10.1186/s12977-017-0369-y (PMC5610415; doi:10.1186/s12977-017-0369-y)

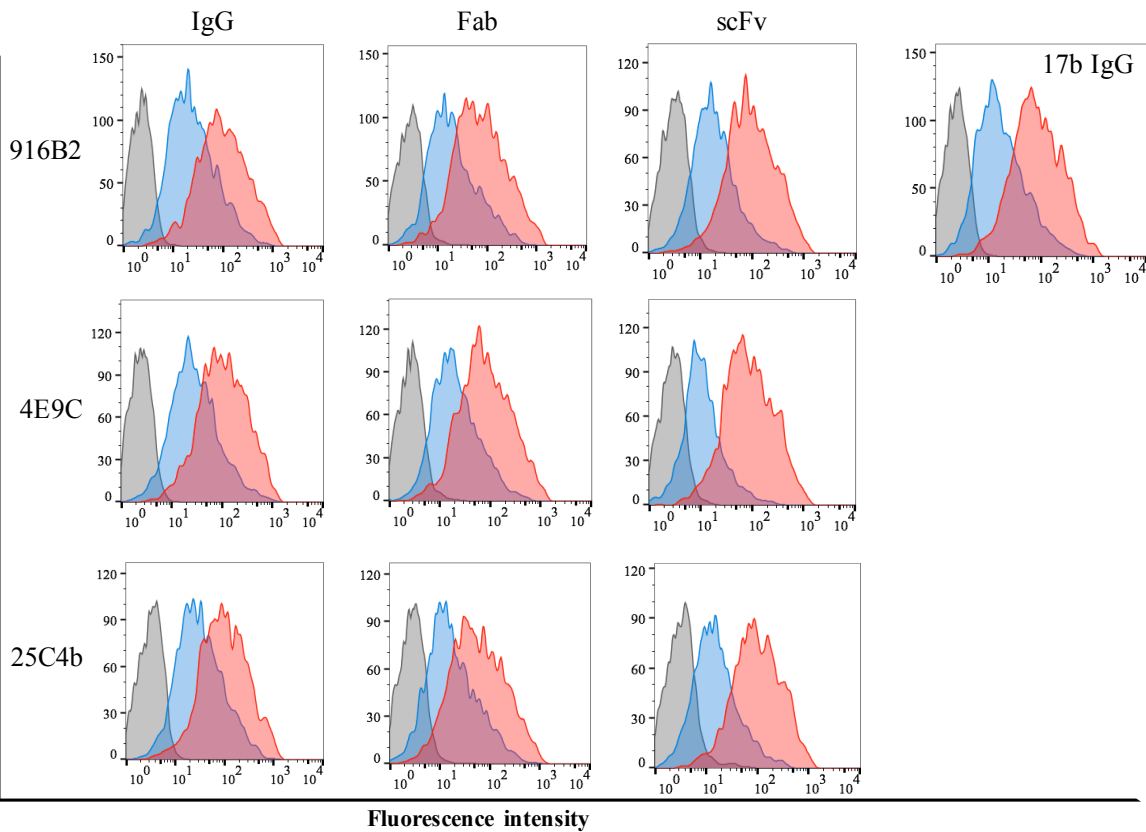

Supplement: Supplementary file 3 — Additional file 3: Figure S1. Binding enhancement of mAbs and their fragments to Env of BaL strain in the presence of sCD4. Binding activity to 293T cells expressing Env of BaL was examined using flow cytometry. Histogram of fluorescence intensity shows the binding of the mAbs as described in Fig. 4. [file 12977_2017_369_MOESM3_ESM.pdf]

Absorbance (405 nm)

917B11

5D6S

12G10

A32

Monomeric gp120 mutants

No sCD4

Ab+sCD4

anti-CD4bs Ab

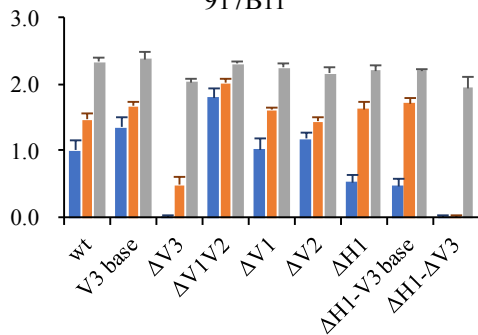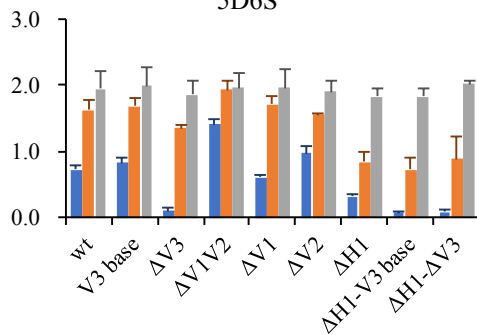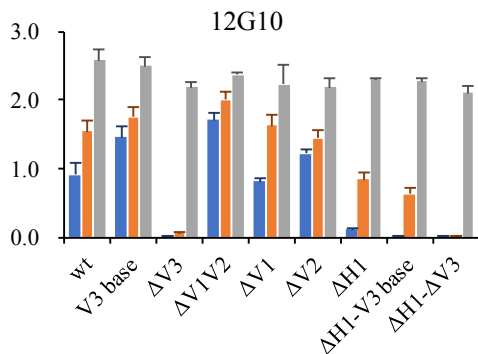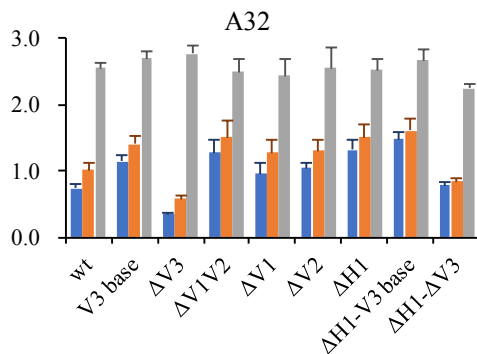

Supplement: Supplementary file 4 — Additional file 4: Figure S2. Binding activities of other anti-CD4i mAbs against gp120 mutants. The binding activities of other anti-CD4i mAbs against gp120 were measured by gp120 capture ELISA. We used the monomeric gp120 of WT and eight mutants as described in Fig. 7. The binding activities of anti-CD4i mAbs in the presence (orange) or absence (blue) of sCD4 and anti-CD4bs mAb, 49G2 (gray) are shown. [file 12977_2017_369_MOESM4_ESM.pdf]

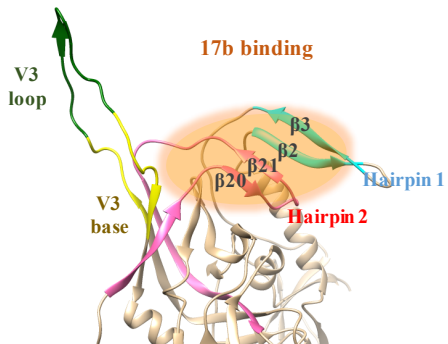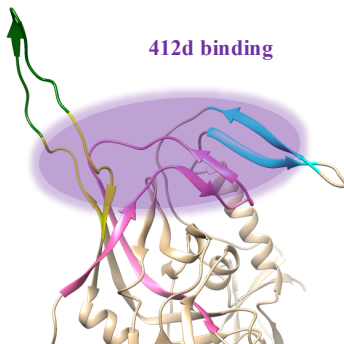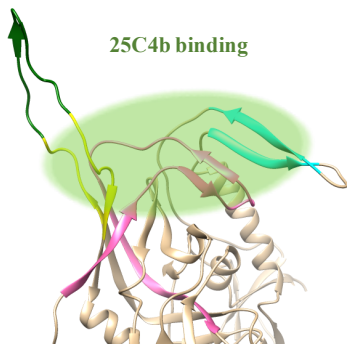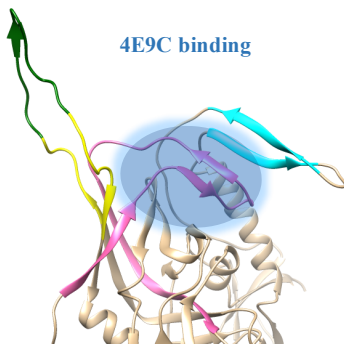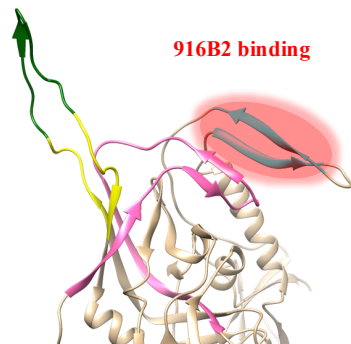

Supplement: Supplementary file 5 — Additional file 5: Figure S3. Comparison of the epitopes of anti-CD4i mAbs. The regions of anti-CD4i mAbs interaction sites were highlighted with each color. The H1 (cyan), H2 (pink), the base of V3 (yellow) and the tip of V3 (green) are shown (PDB accession number 2B4C). 25C4b interacts a region spanning multiple domains of H1 and H2 of the bridging sheet and V3 base as 17b and 412d bindings. 4E9C and 916B2 show the signature bindings against gp120, respectively. [file 12977_2017_369_MOESM5_ESM.pdf]

## a: Heavy chain

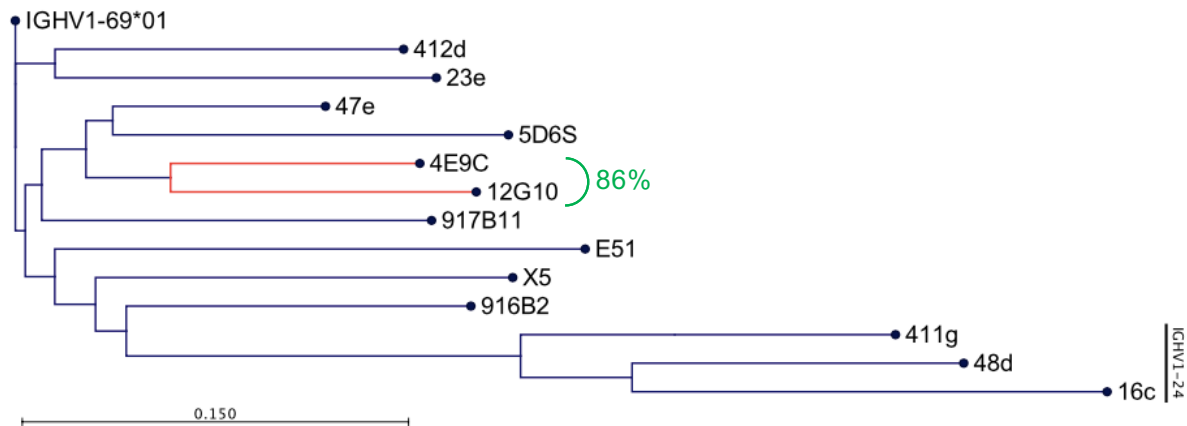

## b: Kappa chain

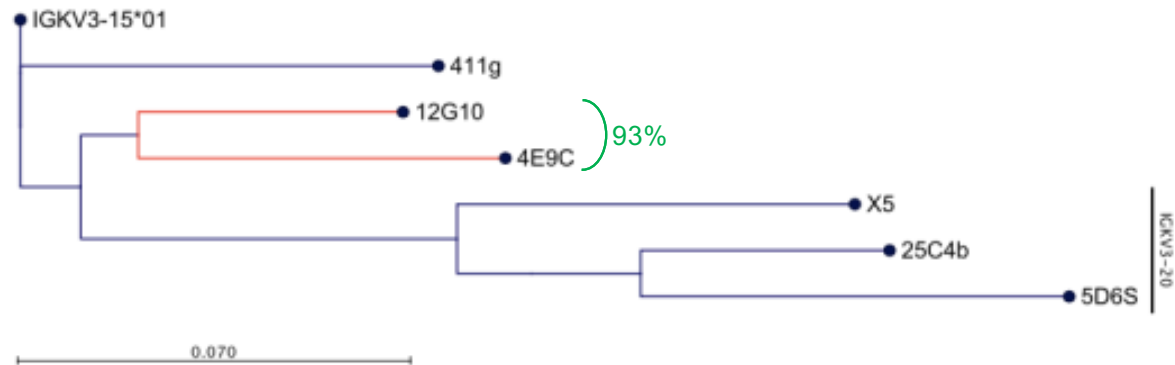

Supplement: Supplementary file 6 — Additional file 6: Figure S4. Phylogenetic analysis of the variable region sequence of 4E9C and 12G10. Variable region sequences of heavy- and kappa-chains were aligned and phylogenetically analyzed. The sequences of anti-CD4i mAbs utilizing IGHV1–69 (412d, 23e, 47e, E51, X5), IGHV1–24 (411g, 48d, 16c), IGKV3–15 (411g) and IGKV3–20 (X5) were obtained from GenBank. The homologies of immunoglobulin genes of heavy- and kappa-chains between anti-CD4i mAbs were determined using Pairwise Sequence Alignment. The same gene usage and high sequence homology between 4E9C and 12G10 are shown (green): 86% for heavy chain and 93% for kappa chain. [file 12977_2017_369_MOESM6_ESM.pdf]
